# Supplementary material for: Development of a Metastatic Uveal Melanoma Prognostic Score (MUMPS) for Use in Patients Receiving Immune Checkpoint Inhibitors
Source: Cancers (Basel). 2021 Jul 20;13(14):3640. doi: 10.3390/cancers13143640 (PMC8306971; doi:10.3390/cancers13143640)
Supplement: Supplementary file 1 [file cancers-13-03640-s001.zip › cancers-1288320-supplementary.pdf]

cancers MDPI

Supplementary

Development of a Metastatic Uveal Melanoma Prognostic Score (MUMPS) for use in Patients Receiving Immune Checkpoint Inhibitors

Deirdre Kelly et. al.

Table S1. Treatment duration and discontinuation in the entire study cohort by treatment groups.

	**Anti-PD1 (*n* = 54)**	**Anti-PD1+anti-CTLA4 (*n* = 21)**	***p*-value**
**Total # cycles Anti-PD1+anti-CTLA4**			-
1	-	1 (5%)	
2	-	6 (29%)	
3	-	7 (33%)	
4	-	7 (33%)	
**Maintenance/Monotherapy**			
Anti-PD1	52 (96%)	9 (43%)	$p < 0.0001$
Anti-PDL1	2 (4%)	0 (0%)	
No maintenance/monotherapy	-	12 (57%)	
**# cycles containing standard dose anti-PD1**			$p = 0.0079$
Average	7.2	3.5	
Median	4	2	
Range	1–38	0-29	
**Reason for discontinuation**			$p = 0.21$
Progression	46 (85%)	15 (71%)	
Toxicity	5 (9%)	4 (19%)	
On-going treatment	1 (2%)	2 (9%)	
Lost to follow-up	2 (4%)	0 (0%)	

Table S2. Treatment and dosing schedule of combination anti-PD-1 + anti-CTLA4.

**Combinations**	**Number of Patients *n* = 21 (%)**
anti-CTLA4 3 mg/kg + anti-PD1 1 mg/kg Q3W, followed by anti-PD1 3 mg/kg Q2W	17 (81%)
Anti-CTLA4 1 mg/kg + anti-PD1 2mg/kg or 200 mg Q3W, followed by anti-PD1 2 mg/kg Q3W	2 (9.5%)
anti-CTLA4 1 mg/kg + anti-PD1 3 mg/kg Q3W, followed by anti-PD1 3 mg/kg Q2W	2 (9.5%)

Abbreviations: Q2W = every 2 weeks; Q3W = every 3 weeks.

Table S3. Genomic analyses of mUM tumor specimens.

	Entire cohort (n = 75)	Anti-PD1 (n = 54)	Anti-PD1 +anti-CTLA4 (n = 21)	p-value
**Impact Genetics**				0.128
Yes	16 (21%)	9 (17%)	7 (33%)	
No	59 (79%)	45 (63%)	14 (67%)	
**NGS**				**0.032**
Yes	50 (67%)	32 (59%)	18 (86%)	
No	25 (33%)	22 (41%)	3 (14%)	
**Impact Genetics**				
**Chromosome 1**				0.163
1p disomy	9 (12%)	5 (9%)	4 (19%)	
1p borderline loss	3 (4%)	1 (2%)	2 (10%)	
1p loss	3 (4%)	2 (4%)	1 (5%)	
Not tested/insufficient	60 (80%)	46 (85%)	14 (67%)	
**Chromosome 3**				0.170
Monosomy 3	15 (20%)	8 (15%)	7 (33%)	
Partial Monosomy	1 (1%)	1 (2%)	0 (0%)	
Not tested/insufficient	59 (79%)	45 (83%)	14 (67%)	
**Chromosome 6p**				**0.049**
6p disomy	12 (16%)	5 (9%)	7 (33%)	
6p gain/borderline gain	3 (4%)	3 (6%)	0 (0%)	
Not tested/ insufficient	60 (80%)	46 (85%)	14 (67%)	
**Chromosome 6q**				0.212
6q disomy	7 (9%)	4 (74%)	3 (14%)	
6q borderline loss	2 (3%)	1 (2%)	1 (5%)	
6q loss	6 (8%)	3 (6%)	3 (14%)	
Not tested/insufficient	60 (80%)	46 (85%)	14 (67%)	
**Chromosome 8p**				0.130
8p disomy	8 (11%)	6 (11%)	2 (10%)	
8p borderline loss	3 (4%)	1 (2%)	2 (10%)	
8p loss	5 (7%)	2 (4%)	3 (14%)	
Not tested/insufficient	59 (79%)	45 (83%)	14 (67%)	
**Chromosome 8q**				0.164
8q gain	14 (19%)	7 (13%)	7 (33%)	

8q partial gain	2 (3%)	2 (4%)	0	
Not tested/insufficient	59 (79%)	45 (83%)	14 (67%)	
**NGS Testing:**				
**BRAF**				0.106
BRAF WT	49 (65%)	32 (59%)	17 (81%)	
BRAF mutated	0	0	0	
Not tested/insufficient	26 (33%)	22 (41%)	4 (19%)	
**NRAS**				0.119
NRAS WT	46 (61%)	30 (56%)	16 (76%)	
NRAS mutated	0	0	0	
Not tested/insufficient	29 (39%)	24 (44%)	5 (24%)	
**GNA11**				0.088
GNA11 209	15 (20%)	11 (20%)	4 (19%)	
GNA11 183	2 (3%)	2 (4%)	0	
GNA11 WT	27 (36%)	15 (28%)	12 (57%)	
Not tested/insufficient	31 (41%)	26 (48%)	5 (24%)	
**GNAQ**				**0.024**
GNAQ 209	21 (28%)	10 (19%)	11 (52%)	
GNAQ 183	1 (1%)	1 (2%)	0	
GNAQ WT	22 (29%)	17 (31%)	5 (24%)	
Not tested/insufficient	31 (41%)	26 (48%)	5 (24%)	
**BAP1**				**0.003**
BAP1 mutated	19 (25%)	8 (15%)	11 (54%)	
BAP1 WT	20 (27%)	15 (28%)	5 (24%)	
Not tested/insufficient	36 (48%)	31 (57%)	5 (24%)	
**SF3B1**				0.062
SF3B1 mutated	6 (8%)	4 (7%)	2 (10%)	
SF3B1 WT	32 (43%)	19 (35%)	13 (62%)	
Not tested/insufficient	37 (49%)	31 (57%)	6 (28%)	

Table S4. Number of patients who received anti-PD1/L1 monotherapy or anti-PD1/L1 + anti-CTLA4 antibodies.

Treatment	MUMPS good risk (score = 3)	MUMPS intermediate risk (score = 2)	MUMPS poor risk (score = 0 or 1)	*p*-value
Anti-PD1 monotherapy	17	28	9	
Anti-PD1 + anti-CTLA4	5	10	6	*p* = 0.522

Table S5. Open Interventional Clinical Trials for the treatment of Metastatic Uveal Melanoma (Current as of 24 January 2021) [1].

Clinical Trial ID	Intervention	Phase	Size[a]	First Posted
NCT04589832	Procaspase-Activating Compound *1 (*PAC-1) in combination with Entrectinib (NTRK Inhibitor)	1b/2	38	Oct 2020
NCT04552223	Nivolumab and Relatlimab (LAG-3 Abtibody)	2	27	Sept 2020
NCT04551352	O7293583, A Tyrosinase-Related Protein 1(TYRP 1) - Targeting CD3 T-Cell Engager	1	310	Sept 2020
NCT04335890	Vaccination with Inhibitor Of Nuclear Factor Kappa B Kinase Subunit Beta (IKKb) matured Dendritic Cells	1	12	April 2020
NCT04283890	Percutaneous hepatic perfusion with Ipilimumab and Nivolumab	1/2	88	Feb 2020
NCT04109456	IN10018, a Focal Adhesion Kinase (FAK) Inhibitor +/- Cobimetinib	1b	52	Sept 2019
NCT03947385	IDE196, a PKC inhibitor+/− Binimetinib in patients with solid tumors harboring GNAQ or GNAQ/11 mutations or PRKC fusions	1/2	217	May 2019
NCT03865212	Intravenous Injection of Vesicular Stomatitis Virus Expressing Human Interferon Beta and Tyrosinase Related Protein 1 (VSV-IFNb-TYRP1)	1	71	Mar 2019
NCT03467516	Tumor Infiltrating Lymphocytes	2	59	Mar 2018
NCT03472586	Immunoembolization in combination with ipilimumab and nivolumab	2	35	Mar 2018
NCT03025256	Intrathecal nivolumab in combination with intravenous Nivolumab in treating patients with leptomeningeal disease	1	50	Jan 2017
NCT03068624	Cyclophosphamide, autologous CD8+ SLC45A2-specific T lymphocytes via hepatic arterial infusion and ipilimumab	1b	30	Mar 2017

[a] Participant size as stated in registry entry.

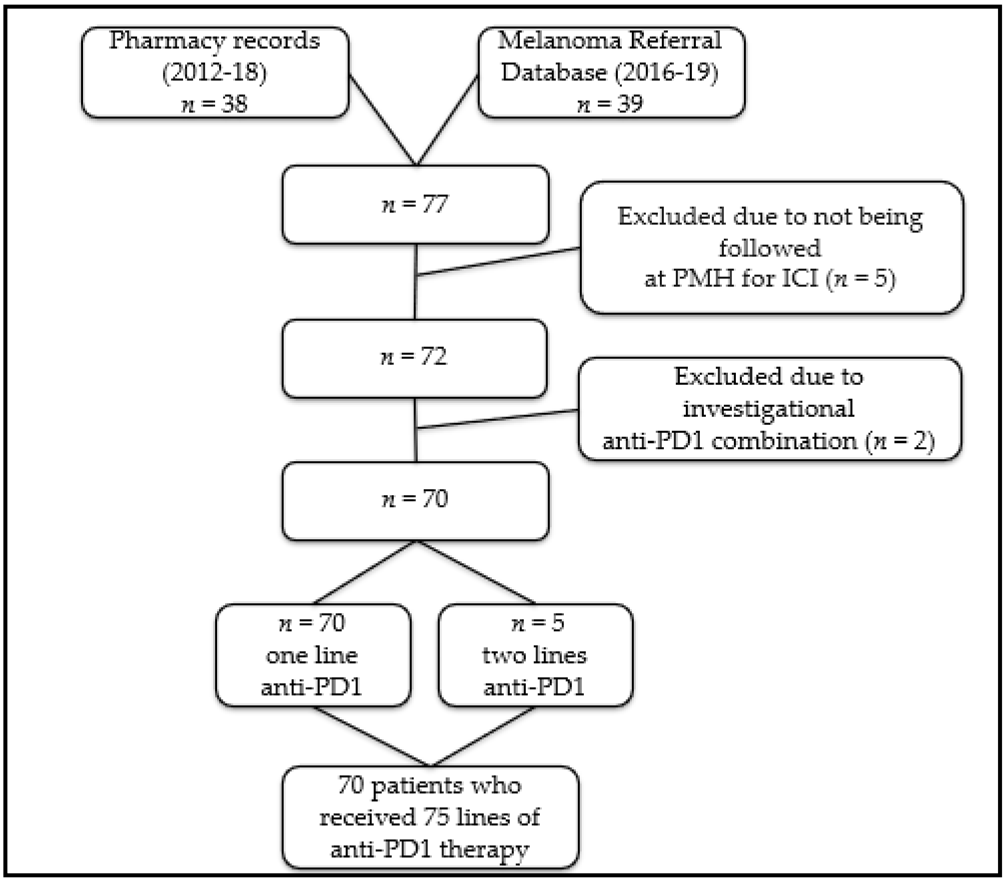

Figure S1. Consort diagram. We identified 77 patients who had received anti-PD1/L1 containing therapy for metastatic uveal melanoma. Two patients were excluded due to non-uveal histology (blue nevus and conjuctival). Two patients were excluded who had received anti-PD1/L1 therapy combined with investigational agents (ie. not anti-CTLA4), Finally, we excluded who were not followed at PM-UHN during their ICI treatment. After exclusion of these patients, the remaining 70 individual patients included 5 patients who had 2 lines of anti-PD1 ICI treatment. We analyzed data from 70 patients who had received a total of 75 lines of anti-PD1 ICI therapy.

References

1. NIH, *Recruiting Studies ; Uveal Melanoma, Metastatic* NIH U.S. National Library of Medicine Clinical Trials.gov URL https://clinicaltrials.gov/ct2/results?cond=Uveal+Melanoma%2C+Metastatic&Search=Apply&recrs=a&age_v=&gndr=&type=&rslt= (Accessed 24 January 2021) 2021.
